# Electromicrobiology: realities, grand challenges, goals and predictions

**DOI:** 10.1111/1751-7915.12400

**Published:** 2016-08-10

**Authors:** Kenneth H. Nealson, Annette R. Rowe

**Affiliations:** ^1^ Department of Earth SciencesUniversity of Southern CaliforniaLos AngelesCAUSA

## Abstract

Electromicrobiology is a subdiscipline of microbiology that involves extracellular electron transfer (EET) to (or from) insoluble electron active redox compounds located outside the outer membrane of the cell. These interactions can often be studied using electrochemical techniques which have provided novel insights into microbial physiology in recent years. The mechanisms (and variations) of outward EET are well understood for two model systems, *Shewanella and Geobacter*, both of which employ multihaem cytochromes to provide an electron conduit to the cell exterior. In contrast, little is known of the intricacies of inward EET, even in these model systems. Given the number of labs now working on EET, it seems likely that most of the mechanistic details will be understood in a few years for the model systems, and the many applications of electromicrobiology will continue to move forward. But emerging work, using electrodes as electron acceptors and donors is providing an abundance of new types of microbes capable of EET inward and/or outward: microbes that are clearly different from our known systems. The extent of this very diverse, and perhaps widely distributed and biogeochemically important ability needs to be determined to understand the mechanisms, importance, and raison d'etre of EET for microbial biology.

## A bit of history

**Figure 1:**
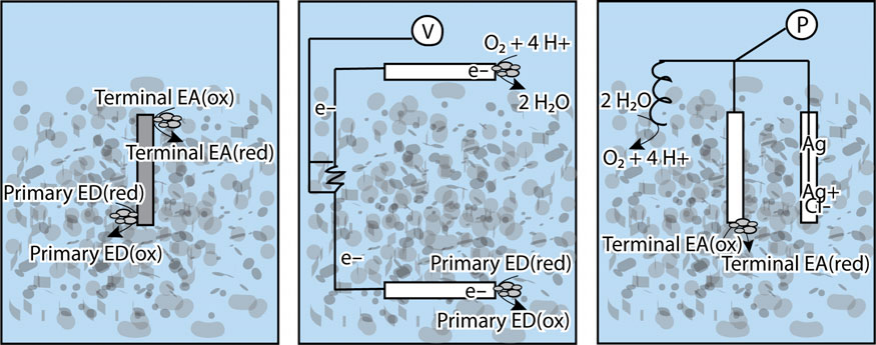
Overview of electrochemical techniques used to study microbial EET including: (A) Oxidation or reduction in insoluble minerals or metals (solid grey bar) analogous to reactions causing corrosion; (B) microbial fuel cell or two electrode (solid white bars) system for quantifying current generation through oxidation of an electron donor (ED) at the anode and reduction in electron acceptor (EA, commonly O_2_) at the cathode and (C) three electrode or half‐cell system for poising electrode potential relative to a reference electrode. A platinum counter electrode, Ag/AgCl reference electrode, and general working electrode (white bar) are illustrated (cathode depicted). Symbols represent: (P) potentiostat and (V) voltmeter.

Electromicrobiology is rapidly morphing into a multidisciplinary area of its own. It was ‘born’ in the early 1900s with experiments showing that microbes could metabolically convert organic carbon into low levels of electricity (Potter, 1911; Cohen, 1931), and revived with the discovery of dissimilatory metal oxide reducing microbes (Lovley and Phillips, 1988; Myers and Nealson, 1988). This included first, the demonstration, of extracellular electron transport (EET), and second, the demonstration that EET‐capable microbes produced moderate levels of electricity in microbial fuel cells (MFCs) (Kim, 1999) (see Logan *et al*., 2006; Lovley, 2006; Rabaey *et al*., 2007, and references therein). This has led to renewed interest in both MFCs and the electrochemical interactions of microbes and minerals. More recently, the reports of electron uptake from insoluble electron donors, including cathodic electrodes, has spurred interest in electrosynthesis (Rabaey and Rozendal, 2010; Rabaey *et al*., 2011; Ross *et al*., 2011), cathodic bioelectrodes for remediation of metal pollution (Hsu *et al*., 2012; Nancharaiah *et al*., 2015), and the growth of autotrophic microbes with electricity as their sole source of energy (Rowe *et al*., 2015). Taken together, these reports provide a window into environmental microbiology that did not exist only a few years ago. The merger of electrochemical approaches, with microbiology and in some cases environmental microbiology (outlined in Fig. 1) has already had a huge impact on understanding microbial physiology and characterizing the diversity of organisms capable of EET.

Taken at a glance, the big picture of how organisms conserve energy remains the same. It is all about electron flow and the use of alternative electron donors and acceptors to fuel life processes; something well known to biochemists and microbiologists. However, electromicrobiology has moved some new and unexpected substrates into the realm of microbial metabolism – substrates that were previously deemed ‘off‐limits’ or ‘non‐edible’ given our previous knowledge of microbial metabolisms. This realization started in 1988, with the reports of two dissimilatory metal reducing microbes in genera that would eventually be named *Shewanella* (Myers and Nealson, 1988; Venkateswaran *et al*., 1999) and *Geobacter* (Lovley and Phillips, 1988). Both of these organisms appeared to defy existing paradigms of electron transfer with their utilization of insoluble metal oxides as electron acceptors. Nearly 30 years of research has given us three proposed models of EET in these canonical systems (described in detail below).

The ability of these EET‐capable bacteria to reduce electrodes (Kim, 1999) has energized the area of MFC work (Logan *et al*., 2006; Lovley, 2006), giving insight into the biological mechanisms underlying empirical observations of anode populations transforming available organic molecules into CO_2_ and electricity. It soon became clear that other bacteria, sometimes the same ones (e.g. *Shewanella*) – could also colonize the cathode and form active biofilms, catalysing electron transfer from the cathode to oxygen or other soluble electron acceptors (Ross *et al*., 2011; Hsu *et al*., 2012). Thus, EET is not a one‐way street, but bi‐directional, with many bacteria now known that are capable of inward‐, others of outward‐ and some both directions of EET (Rabaey and Rozendal, 2010; Rabaey *et al*., 2011; Ross *et al*., 2011; Rowe *et al*., 2015; Ishii *et al*., 2015). And though there are several reports of EET‐capable microbes that utilize solid substrates as an electron source (Rabaey and Rozendal, 2010; Ross *et al*., 2011; Rowe *et al*., 2015), mechanistically we know very little about how these organisms obtain electrons from solid substrate electron donors.

## The realities: what we know

In *Shewanella* and *Geobactor*, EET was originally perceived as a mechanism for the transport of electrons to the cell exterior (across the periplasm and outer cell membrane) to allow for the conversion of electrochemical energy into biological energy. Alternatively, for fermentative microbes, EET can be viewed as a method for maintaining redox balance (i.e. regenerating NAD^+^) while predominantly making ATP by substrate level phosphorylation. Fermentatively, the ‘problem’ solved by EET is that it allows electron flow to continue in the absence of the usual soluble electron acceptors, and without the accumulation of hydrogen or other reduced fermentation end‐products. Energetically, if and/or how the reverse process of EET (electron uptake from solid substrates), allows organism to conserve energy has yet to be studied in great detail though growth using iron minerals and solid state electrodes has been reported (Rowe *et al*., 2015; Ishii *et al*., 2015). The physiological implications of oxidative‐EET metabolisms, as well as more mechanistic understanding of novel EET processes are currently being investigated.

## Multi‐haem cytochromes and cytochrome networks are keys

**Figure 2:**
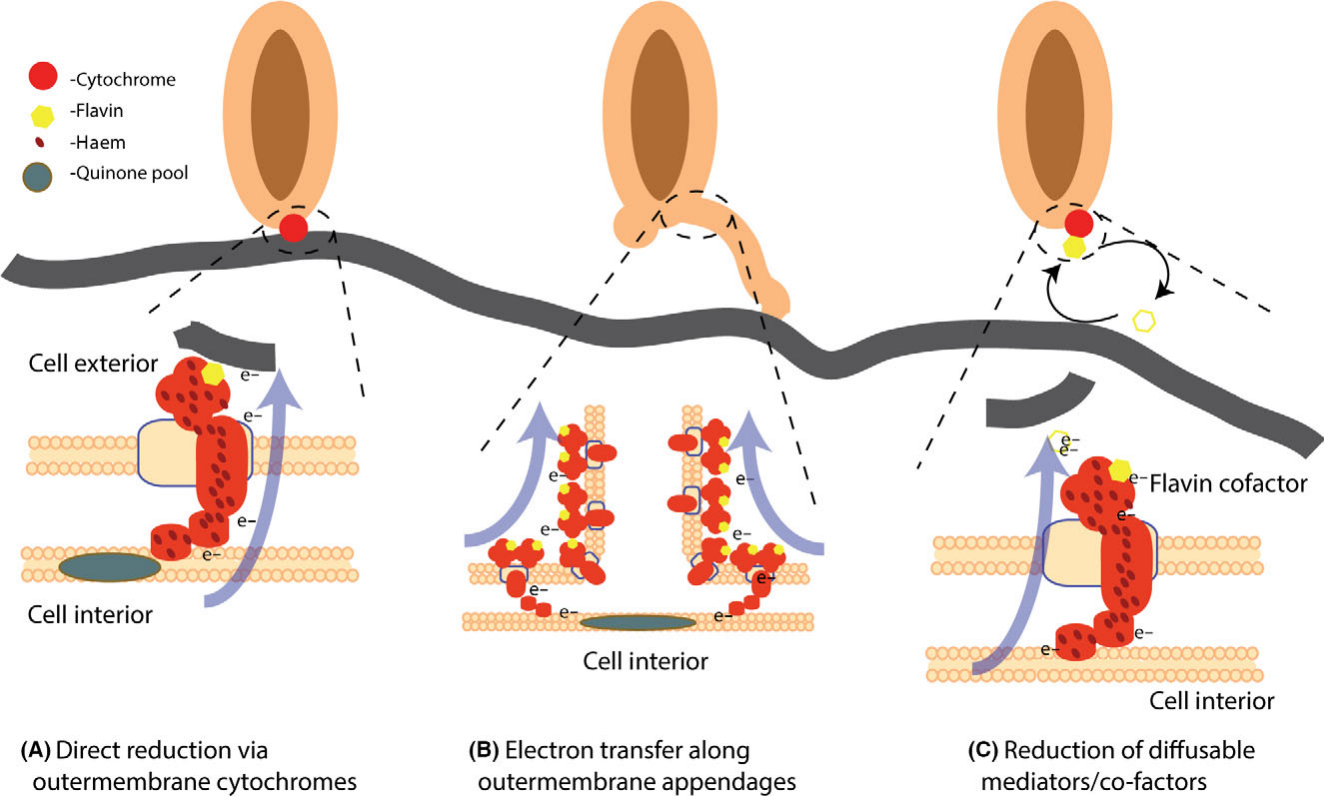
Schematic overview of the major modes of extracellular electron transfer (EET) as utilized by *Shewanellla oneidensis* MR‐1.

*Shewanella* and *Geobacter*, the two best studied organisms in electrobiology, employ similar approaches to move electrons to and across the outer membrane (illustrated for *Shewanella* in Fig. 2). However, these modes differ with regard to the details of the specific proteins involved and/or interactions with other cellular features. In both organisms, a series of multihaem c‐type cytochromes act together to provide a ‘conduit’ to the multi‐haem protein complexes on the outer membrane that directly reduce solid substrates (Hartshorne *et al*., 2009; Clarke *et al*., 2011), as well as some soluble substrates that form solids upon reduction (e.g. U(VI)). Once the electrons are transported to the cell surface, other variations on the theme occur: (i) electron donation occurring directly from the cytochrome network to solid substrates (Richardson *et al*., 2012; Liu *et al*., 2014); (ii) electron transport over considerable distances via conductive cellular appendages or nanowires (El‐Naggar *et al*., 2008, 2010) or (iii) transfer to redox‐active electron shuttles (Gralnick, 2012; Gross and El‐Naggar, 2015) (Fig. 2). In *Shewanella* and *Geobacter*, several multi‐haem cytochromes were identified as participants in EET using genetic techniques (see review by Fredrickson *et al*., 2008; Shi *et al*., 2009), X‐ray crystallography of several of these multi‐haem cytochromes have revealed an arrangement of haems (Shi *et al*., 2009) that provide an ‘electron hopping’ pathway across the cell membrane (Pirbadian and El‐Naggar, 2012; Byun *et al*., 2014) to an outer membrane protein complex that provides the ability for direct electron transfer to solid substrates (Richardson *et al*., 2012; Liu *et al*., 2014). Considering that: (i) flavins (riboflavin or flavin mononucleotide) bind to these proteins, changing their redox potentials (Okamoto *et al*., 2013, 2014a,b; Edwards *et al*., 2015; Xu *et al*., 2016); (ii) flavins and other quinone‐type molecules at high concentration may serve as electron shuttles to more distant electron acceptors (Gralnick, 2012) and (iii) conductive nanowires may carry electrons to more distant electron acceptors (Gorby *et al*., 2006; El‐Naggar *et al*., 2010), one begins to see the potential metabolic broadening that EET provides the microbial world (El‐Naggar and Finkel, 2013).

## There is a diversity of electron transfer interactions

Recent discoveries in electromicrobiology have pointed to the fact, we are only ‘scratching the surface’ in terms of understanding microbe–electrode interactions, with important implications to environmental microbiology. In marine sediments, it has been demonstrated that so‐called ‘cable bacteria’ can provide a conductive link between anoxic and oxic levels of stratified sediments; adding, quite literally, a new (cm level) dimension to the potential impact of electron transfer reactions (Pfeffer *et al*., 2012; El‐Naggar and Finkel, 2013). How this spatially uncoupled redox process provides energy to a whole population chain of cells remains to be seen, but has expanded the spatial scale of electron transfer reactions. In addition, EET is clearly not limited to organisms of the same species. Several reports have suggested that microbes can transfer electrons to metabolic partners in syntrophic reactions (Summers *et al*., 2010; Rotaru *et al*., 2014; McGlynn *et al*., 2015; Wegener *et al*., 2015). In several, cases we are also broadening the taxonomic breadth, as well as structural variation in organisms capable of EET (Gram‐positive, Archaeal cell walls, etc.), which further highlights the potential diversity of EET mechanisms that have yet to be discovered (Rowe *et al*., 2015). There may be even more EET variations; for example, incorporation of melanin as a conductive element (Turick *et al*., 2003) and use of cell‐bound minerals as electron carriers (Kato *et al*., 2010; Nakamura *et al*., 2010, 2013), and others, waiting to be discovered.

## Not all microbe–electrode interactions are direct

Mediators, diffusible molecules that can shuttle electrons from cells to distant terminal electron acceptors have always been proposed as a mechanisms for how organisms can interact with electrodes, and in fact maybe the exclusive mode of electron transfer for some species (i.e. *Psuedomonas aeruginosa*) (Gralnick and Newman, 2007; Wang *et al*., 2010). However, a different mechanism of current generation in microbial systems, through the reactivity of extracellular enzymes and electrodes and/or reduced minerals has recently been reported (Deutzmann *et al*., 2015). In this case, electrode–enzyme interactions with hydrogenases generate hydrogen, producing cathodic currents during the process, with the hydrogen ultimately microbially consumed. This report emphasizes that not all electrochemically active microbes utilize EET, and that careful controls and electrochemical tests are required to more thoroughly investigate mechanisms.

## Current models of EET are still debated

Even in the intensely studied model systems, there are still debates as to the ecophysiological relevance of the proposed modes of EET in both *Geobacter* and *Shewanella*. In *Shewanella*, several results point to the dominant mode of electron transfer being redox conductivity via electron hopping through a cytochrome network (El‐Naggar *et al*., 2008; Pirbadian and El‐Naggar, 2012; Pirbadian *et al*., 2014). In *Geobacter*, the mechanism behind electron transfer reactions is being debated. It has recently been challenged that electron transfer in biofilms is likely dominated (or limited) by redox conductivity (Yates *et al*., 2015, 2016) as opposed metallic‐like conductivity which has also been proposed (Malvankar *et al*., 2011).

The role that flavins play in electron transfer reactions has also been widely debated. In *Shewanella*, flavins have been reported to be important, both as electron shuttles (Marsili *et al*., 2008; Kotloski and Gralnick, 2013) and as bound cofactors (Okamoto *et al*., 2013, 2014a,b; Xu *et al*., 2016). Although still up for debate, the field seems to be converging on a consensus in terms of what mechanisms dominate and or how the various models for electron transfer fit together, and/or under what physiological conditions.

## Grand challenges – what we don't know

Almost thirty years of work by several laboratories has led to a wealth of knowledge regarding both *Shewanella* and *Geobacter*, with hundreds of publications on the distribution of these organisms, the genes and the proteins they encode that are key to successful EET, and characterization of the mechanism(s) involved. Despite the excellent work that has appeared, controversies and unknowns remain, including: (i) the role(s) of flavins (FMN and RF) in EET; (ii) the mechanism(s) of EET, whereby bacterial nanowires conduct electrons over large distances; (iii) the mechanisms whereby EET into the cells occurs; (iv) mechanism(s) whereby microbes sense, and respond to surface charge, and changes in surface charge; (v) the taxonomic and mechanistic diversity of EET in the microbial (and perhaps eukaryotic) world and (vi) the *raison d'etre* of EET – what are the ecological uses for, and ramifications of, the ability to transfer electrons outside the cell, sometimes for long distances.

Given the state of the field now, one might say that we are at an intellectual crossroad: one not unfamiliar to those of us who ‘grew up’ with the ‘*Escherichia*,* Salmonella*,* Bacillus*’ mafia. Electromicrobiology is dominated by the two model systems, *Shewanella* and *Geobacter*, with sequenced genomes, hundreds of mutants and a huge backlog of knowledge and information. Several of the challenges listed above (i‐iv), will be dealt with in the coming few years (in fact, are being worked on as you read). While they are of great interest and importance, they fall more in the category of incremental knowledge: questions that can best be addressed with all of the advantages of the model systems.

In our opinion, the grand challenge is the elucidation of the breadth and diversity of EET, both taxonomically and functionally in environment. This includes the attainment of EET relevant molecular/genetic markers, of which we have only limited insight from the current model systems. How do microbes sense and respond to surface potential? What are the genes and proteins that can be used to define the distribution of EET? Can EET be used as a sensing and/or communication system for microbes? Our current understanding limits our appreciation for the role(s) of EET in microbiology: intercellular energy transfer, intercellular information transfer, microbial syntrophy and symbioses, etc. In terms of mechanisms, recent reports of Gram‐positive *Firmicutes* that are capable of EET (Wrighton *et al*., 2011) (and which must be mechanistically different), as well as reports of Gram‐negative microbes that appear to lack homologous multi‐haem cytochromes used by *Shewanella* or *Geobacter* provide guideposts for exciting work to advance our understanding of the biology and ecology of electromicrobiology.

## Goals and predictions

What will it take to face up to this grand challenge? First, microbiologists who understand electrochemistry, and electrochemists who understand microbiology. This is no small task, but given the potential importance of this area in microbial ecology, it is one that should be taken seriously.

Second, this is the type of work that is not easily funded. It is comparatively easy to advance knowledge in a field, where there is already enough information to design sophisticated tests of elegant hypotheses, as can be done in the worlds of *Shewanella* and *Geobacter*. It is far more difficult to fund discovery science, when the hypothesis is: ‘diversity is expected, and it will be worth knowing about’! On the basis of early results of such efforts, we believe that the hypothesis is already proven, and that new strains will soon become household names making it apparent that EET‐capable microbes are common in nearly all environments, and doing things that no one told us about when we were students!
